# *Streptococcus Pneumoniae* Bacteremia with Acute Kidney Injury and Transient ADAMTS13 Deficiency

**DOI:** 10.1155/2023/3283606

**Published:** 2023-04-29

**Authors:** Sam Van Hove, Alexis Werion, Ahalieyah Anantharajah, Leila Belkhir, Marie-Astrid van Dievoet, Philippe Hantson

**Affiliations:** ^1^Department of Intensive Care, Cliniques Universitaires Saint-Luc, 1200 Brussels, Belgium; ^2^Institut de Recherche Expérimentale et Clinique (IREC), Université Catholique de Louvain, Neuve, Belgium; ^3^Laboratory of Microbiology, Cliniques Universitaires Saint-Luc, 1200 Brussels, Belgium; ^4^Department of Internal Medicine and Infectious Diseases, Cliniques Universitaires Saint-Luc, 1200 Brussels, Belgium; ^5^Laboratory of Hemostasis, Cliniques Universitaires Saint-Luc, 1200 Brussels, Belgium; ^6^Louvain Centre for Toxicology and Applied Pharmacology, Université Catholique de Louvain, 1200 Brussels, Belgium

## Abstract

A 43-year-old woman with a medical history of splenectomy for immune thrombocytopenic purpura was diagnosed with *Streptococcus pneumoniae* bacteremia. Her initial complaints were fever and more importantly painful extremities that appeared cyanotic. During her hospitalisation, she never developed cardiocirculatory failure but presented acute kidney injury (AKI) with oliguria. Laboratory investigations confirmed AKI with serum creatinine 2.55 mg/dL which peaked at 6.49 mg/dL. There was also evidence for disseminated intravascular coagulation (DIC) with decreased platelet count, low fibrinogen levels, and high D-dimer levels. There were no signs of haemolytic anaemia. The initial ADAMTS13 activity was low (17%) but slowly recovered. Renal function progressively improved with supportive therapy, as opposed to the progressing skin necrosis. The association of DIC and low ADAMTS13 activity may have contributed to the severity of microthrombotic complications, even in the absence of thrombotic microangiopathy as thrombotic thrombocytopenic purpura (TTP) or pneumococcal-associated haemolytic uremic syndrome (pa-HUS).

## 1. Introduction

Thrombotic microangiopathy (TMA) is a group of diseases characterized by nonimmune microangiopathic haemolytic anaemia, thrombocytopenia, and organ failure (e.g. acute kidney injury). TMA includes different subgroups like thrombotic thrombocytopenic purpura (TTP), typical haemolytic uremic syndrome (HUS) commonly triggered by Shiga-like toxin-producing*Escherichia coli*, atypical haemolytic and uremic syndrome (aHUS), a genetically or acquired complement dependant disease, and HUS secondary to other conditions such as invasive *Streptococcus pneumoniae* infection [1, 2]. This rare complication occurs mainly in neonates and children up to the age of five. It is exceptional in adults. But a history of splenectomy can be a possible risk factor [3–5]. The determination of ADAMTS13 activity in plasma could be helpful to specify the type of TMA as ADAMTS13 activity is typically <10% in thrombotic thrombocytopenic purpura (TTP), in contrast to HUS or aHUS. We present a case of invasive *S. pneumoniae* infection complicated by peripheral gangrene and acute kidney injury (AKI) in the absence of septic shock, and we discuss the significance of a transient ADAMTS13 deficiency for the differential diagnosis between TMA and sepsis-related disseminated intravascular coagulation (DIC) with thrombotic complications that include acute kidney injury.

## 2. Case Report

**Figure 1:**
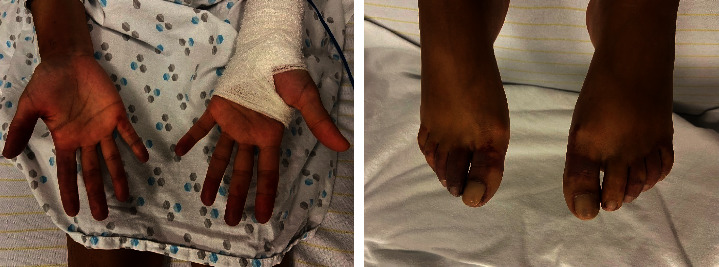
Cyanotic and ischaemic features of the extremities on admission.

A 43-year-old woman was presented at the emergency department with symptoms of fever (up to 41°C), vomiting, and diarrhoea. Her chief complaint was pain in the extremities, in the distal parts of her fingers and toes. She has a past medical history of splenectomy at the age of 18 years for immune thrombocytopenic purpura. Vaccination for pneumococcal infection was adequately performed only over a few years after splenectomy. However, according to the medical records, her last vaccination was administered more than 5 years ago. On admission to the emergency department, she presented with the following vital signs: temperature 36.3°C, heart rate 94/min, arterial blood pressure 111/74 mmHg, respiratory rate 26/min, and pulse oxygen saturation (SpO_2_) at ambient air 99%. At physical examination, the extremities of both fingers and toes appeared symmetrically cyanotic and were extremely painful (Figure 1). Laboratory investigations revealed the following: CRP 291 mg/L (<5.0), haemoglobin 14.6 g/dL (12.2–15.0), white blood cell count 17,820·10^3^/*μ*L, platelet count 63·10^3^/*μ*L, INR 1.99 (0.80–1.20), fibrinogen 82 mg/dL (150–450), D-dimers >35000 ng/mL (<500), von Willebrand factor (VWF) antigen 200% (50–150), ADAMTS13 activity 17% (>40), serum creatinine 2.55 mg/dL (0.60–1.30), LDH 783 IU/L (<250), total bilirubin 1.1 mg/dL (<1.2), schistocytes <1%, Coombs direct test (−), haptoglobin 1.76 g/L (0.30–2.0), complement C3 0.92 g/L (0.90–1.80), complement C4 0.32 g/L (0.10–0.40), antineutrophil cytoplasmic antibodies (−), anticardiolipin antibodies (−), and cryoglobulin detection (−). Urinalysis showed: red blood cells 5/HPF, white blood cells 8/HPF, no casts, and proteinuria 0.83 g/g of urine creatinine (<0.2) including albuminuria of 316 mg/g (<30). The evolution of the laboratory results is summarised in Table 1.

**Figure 2:**
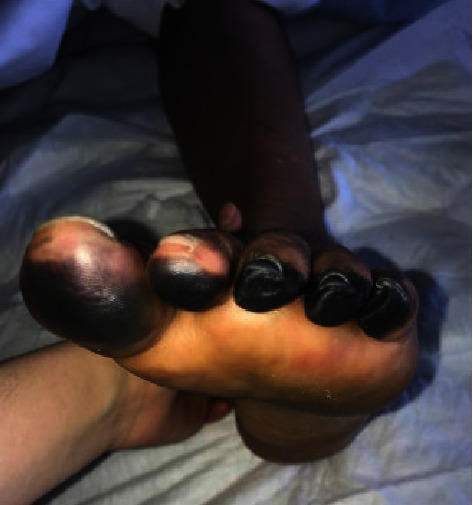
Evolution of peripheral necrosis (left foot, day 10).

The bacterial growth was detected in the four aerobic and anaerobic blood cultures drawn on hospital admission, after 9 hours of incubation. *S. pneumoniae* was detecte, and could later be identified as belonging to serogroup 24. The isolate was resistant to erythromycin, minocycline, and clindamycin. It had reduced susceptibility to penicillin (MIC = 0.5 mg/L), categorised as “susceptible with increased exposure” according to EUCAST clinical breakpoints (Table 2). Bacteremia was treated by intravenous administration of ceftriaxone (4 g daily) for 2 weeks. The patient never developed cardiocirculatory shock nor other end-organ failures except for the acute kidney injury. The patient's renal function recovered progressively, and she did not require hemodialysis. Unfortunately, there was a progression of dry skin necrosis of the toes, bilaterally (Figure 2), and a distal amputation will probably be required. The patient left the hospital two months later with a rehabilitation program. Vaccination against *Haemophilus influenza B, Meningococcus* A, B, C, W, Y, and *S. pneumoniae* was administered one month later.

## 3. Discussion

Invasive pneumococcal disease (IPD) incidence has significantly decreased over the last decades, through the use of pneumococcal conjugate vaccines (PCV), yet it remains a global health issue. Especially, non-PCV serotypes remain a matter of concern, as illustrated by this serotype 24 isolate which is not included in the 23-valent pneumococcal polysaccharide vaccine. Serotype 24F has been described as an important emerging serotype with high invasiveness and is associated with antimicrobial resistance. Penicillin, macrolide-lincosamide, and tetracycline resistance have been reported [6].

Acute kidney injury is a possible complication of *S. pneumoniae* bacteremia, even in the absence of shock. In older reports, the nature of kidney injury was only noted as “nephritis” and was mainly reported following pneumonia affecting young children [7]. In 1970, Schenk et al. described three cases of pneumococcal bacteremia associated with thrombocytopenia and glomerular and arteriolar thrombosis without mentioning haemolytic anaemia or HUS [8]. Recently, the association of *S. pneumoniae* with HUS was described in young children and was usually associated with a very poor clinical outcome [3, 9, 10]. Serotypes 14, 6B, 9V, 19, 3, 8, 23F, and 19A appear particularly associated with HUS [3]. In the context of pneumococcal infection, it appears important to achieve an accurate diagnosis of the nature of kidney injury as it may directly influence both management and prognosis. Acute tubular necrosis occurs in patients with septic shock and DIC. Immune-complex-mediated acute glomerulonephritis is a rare complication of pneumococcal infection in adults [11]. It may be difficult to differentiate HUS from DIC as both may present with microangiopathic haemolytic anaemia, thrombocytopenia, and renal injury or failure. In HUS, fibrinogen levels, prothrombin, and partial thromboplastin times are typically normal or slightly elevated. In addition, in *S. pneumoniae*-associated HUS, a positive Coombs test was found in up to 90% of children; complement consumption also appears to be common [12]. Our patient had no biological criteria for haemolytic anaemia and laboratory results were consistent with DIC. The severity of cutaneous lesions and kidney injury was also interpreted as being mainly the consequence of DIC, as the patient did never develop shock nor required vasopressors.

Deficiency of the von Willebrand factor (VWF)-cleaving protease, ADAMTS13 (a disintegrin-like metalloprotease with thrombospondin type 1 repeats), is found in most patients with TTP, and this deficiency is suspected to cause platelet aggregation and microthrombi formation in the circulation, developing typical TMA [13]. Deficiency of ADAMTS13 in patients with TTP is caused by genetic defects or by autoantibodies inhibiting its function. ADAMTS13 activity is typically <10% in TTP, in contrast to the unchanged or moderately decreased ADAMTS13 activity in HUS [14]. In the present observation, the initial ADAMTS13 activity was low (17%), no inhibitor was found, and progressive recovery of ADAMTS13 activity was observed over time. The relation between reduced ADAMTS13 activity and DIC is currently not clearly determined and can be a matter of discussion [15]. ADAMTS13 activity has also been shown to decrease in other conditions than TTP, following severe malaria, sepsis, or DIC, although its pathogenic role appears less clear than in TTP [16–19]. The pathophysiological mechanism may involve reduced enzyme synthesis, enzyme inactivation by thrombin, and consumption of ADAMTS13 by the release of ultralarge VWF mediated by inflammatory factors [20]. Exceptionally, the presence of a strong inhibitor of ADAMTS13 activity was reported in two patients with an infection-related severe multiorgan dysfunction (including renal failure) who presented laboratory features of both DIC and TTP [21]. The decrease in ADAMTS13 activity was also investigated as a marker of sepsis severity and was demonstrated to reflect the severity of endothelial injury [22, 23]. ADAMTS13 activity, which was in this setting, mildly decrease compared to TTP, but this phenomenon was associated with a poorer prognosis in patients presenting DIC [21, 24]. In a series of septic patients with DIC, Ono et al. found that the incidence of isolated renal failure was higher in those patients with ADAMTS13 activity <20%, with a strong correlation between ultralarge VWF multimers and creatinine levels [25]. This is also supported by other studies showing a higher incidence of renal failure in patients with deficient ADAMTS13 and/or high VWF propeptide levels [17, 22, 26]. Finally, a transient ADAMTS13 deficiency has been observed in some patients with pneumococcal-associated HUS [27].

We concluded that our patient did not present a typical pneumococcal-associated HUS, but the extensive digital necrosis and the acute, reversible, and renal injury resulted from a sepsis-induced DIC with secondary severe ADAMTS13 deficiency. This case illustrates that, although the mechanisms of severe ADAMTS13 deficiency in sepsis are different from those of idiopathic TTP, the clinical features of patients with sepsis-induced DIC and severe ADAMTS13 deficiency are similar to those of patients with idiopathic TTP. To date, it seems too early to recommend ADAMTS13 supplementation in this indication [25–28].

## Figures and Tables

**Table 1:** Evolution of laboratory data.

	Day 1	Day 2	Day 3	Day 4	Day 5	Day 6	Day 8	Day 9	Day 18
Platelet (×10³/*μ*L)	63	87	55	59	38	55	257	380	884
D-dimers (ng/mL)	>35000	—	—	—	—	3610	—	—	—
Fibrinogen (mg/dL)	82	127	416	338	—	—	—	—	—
ADAMTS13 (%)	17	—	29	—	—	—	—	35	45
Creatinine (mg/dL)	2.55	3.58	4.75	5.87	6.49	7.50	4.54	2.79	1.15
Haptoglobin (g/L)	—	1.76	—	—	—	—	—	3.18	—
LDH (IU/L)	783	892	370	436	417	319	275	264	175
CK (IU/L)	175	307	1821	3552	3184	1510	420	—	65
CRP (mg/dL)	291	409.1	313.6	176.4	161	59.4	94.3	89.1	56.1

**Table 2:** Antimicrobial susceptibility of *Streptococcus pneumoniae* isolates using disk diffusion and E-test gradient method.

Antimicrobial agent	Interpretation^1^
Oxacillin	Screen positive^2^
Benzylpenicillin	Susceptible, increased exposure (MIC 0.5 mg/L)
Ceftriaxone	Susceptible (MIC 0.125 mg/L)
Clindamycin	Resistant
Trimethoprim-sulfamethoxazole	Susceptible
Erythromycin	Resistant
Minocycline	Resistant
Moxifloxacin	Susceptible
Vancomycin	Susceptible

^1^Antimicrobial susceptibility to the different antimicrobial agents was determined and interpreted according to EUCAST clinical breakpoints version 12.0 breakpoint. ^2^Beta-lactam resistance detected (according to the EUCAST clinical breakpoints version 12.0 *Streptococcus pneumoniae*: Flow chart based on the oxacillin screen test for beta-lactam resistance mechanisms).

## Data Availability

The data supporting the findings of this study are available from the corresponding author upon request.
